# Supplementary material for: Artificial intelligence literacy, sustainability of digital learning and practice achievement: A study of vocational college students
Source: PLoS One. 2025 Oct 1;20(10):e0332175. doi: 10.1371/journal.pone.0332175 (PMC12488029; doi:10.1371/journal.pone.0332175)
Supplement: Attachment — Submitted filename: PONE-D-25-07315_reviewed.pdf [file pone.0332175.s002.pdf]

PLOS ONE

Artificial Intelligence Literacy, Sustainability of Digital Learning and Practice Achievement: A Structural Equation Model

--Manuscript Draft--

Manuscript Number:	PONE-D-25-07315
Article Type:	Research Article
Full Title:	Artificial Intelligence Literacy, Sustainability of Digital Learning and Practice Achievement: A Structural Equation Model
Short Title:	AI,SDLC&Practice,A SEM Model
Corresponding Author:	Guangyu Xu, Dr<br>East China Normal University<br>Jilin City, CHINA
Keywords:	AI literacy; Sustainable digital learning competences; SEM; Practice achievement; COM-B; vocational higher education
Abstract:	The rise of artificial intelligence in the evolving world of information and communication technologies is reflected in profound educational changes. Higher education has been at the forefront of the changes, but vocational higher education does not seem to have received enough attention. Focusing on artificial intelligence literacy and sustainability of digital learning competences, this study examined the operational behaviors of 1004 students in a practical course in a higher education institution in mainland China by using the COM-B model. The results showed that AI literacy was positively correlated with sustainable digital learning ability, and AI literacy was positively correlated with students' sustainable digital learning behaviors through sustainability of digital learning competences. However, students in vocational institutions are not able to translate this learning behavior into a path to achieve good performance in practice, and even sustainability of digital learning competences can be slightly counterproductive. There is no excessive effect on the model after controlling for variables such as gender, family resources, and ethnic minorities, which contributes to SDG5 educational equity.
Order of Authors:	Xuefei Lin, PH.D
	Guangyu Xu, Dr
	Bin Xiong
Additional Information:	
Question	Response

Powered by Editorial Manager® and ProduXion Manager® from Aries Systems Corporation

Financial Disclosure<br><br>Enter a financial disclosure statement that describes the sources of funding for the work included in this submission. Review the submission guidelines for detailed requirements. View published research articles from PLOS ONE for specific examples.<br><br>This statement is required for submission and will appear in the published article if the submission is accepted. Please make sure it is accurate.	Yes

Funded studies<br>Enter a statement with the following details:<br>• Initials of the authors who received each award<br>• Grant numbers awarded to each author<br>• The full name of each funder<br>• URL of each funder website<br>• Did the sponsors or funders play any role in the study design, data collection and analysis, decision to publish, or preparation of the manuscript?<br><br>Did you receive funding for this work?	

Powered by Editorial Manager® and ProduXion Manager® from Aries Systems Corporation

Please add funding details.<br>as follow-up to "Financial Disclosure<br><br>Enter a financial disclosure statement that describes the sources of funding for the work included in this submission. Review the submission guidelines for detailed requirements. View published research articles from PLOS ONE for specific examples.<br><br>This statement is required for submission and will appear in the published article if the submission is accepted. Please make sure it is accurate.<br><br>Funded studies<br>Enter a statement with the following details:<br>• Initials of the authors who received each award<br>• Grant numbers awarded to each author<br>• The full name of each funder<br>• URL of each funder website<br>• Did the sponsors or funders play any role in the study design, data collection and analysis, decision to publish, or preparation of the manuscript?<br><br>Did you receive funding for this work?"	This research was funded by a grant from the Shanghai Key Laboratory of Pure<br>Mathematics and Mathematical Practice, grant number 18DZ2271000 By XiongBin
Please select the country of your main research funder (please select carefully as in some cases this is used in fee calculation).	CHINA - CN

Powered by Editorial Manager® and ProduXion Manager® from Aries Systems Corporation

as follow-up to "Financial Disclosure<br><br>Enter a financial disclosure statement that describes the sources of funding for the work included in this submission. Review the submission guidelines for detailed requirements. View published research articles from PLOS ONE for specific examples.<br><br>This statement is required for submission and will appear in the published article if the submission is accepted. Please make sure it is accurate.<br><br>Funded studies<br>Enter a statement with the following details:<br>• Initials of the authors who received each award<br>• Grant numbers awarded to each author<br>• The full name of each funder<br>• URL of each funder website<br>• Did the sponsors or funders play any role in the study design, data collection and analysis, decision to publish, or preparation of the manuscript?<br><br>Did you receive funding for this work?"	

Powered by Editorial Manager® and ProduXion Manager® from Aries Systems Corporation

Competing Interests<br><br>Use the instructions below to enter a competing interest statement for this submission. On behalf of all authors, disclose any competing interests that could be perceived to bias this work—acknowledging all financial support and any other relevant financial or nonfinancial competing interests.<br><br>This statement is required for submission and will appear in the published article if the submission is accepted. Please make sure it is accurate and that any funding sources listed in your Funding Information later in the submission form are also declared in your Financial Disclosure statement.	The authors have declared that no competing interests exist.

View published research articles from PLOS ONE for specific examples.<br><br>NO authors have competing interests<br><br>Enter: The authors have declared that no competing interests exist.<br><br>Authors with competing interests<br><br>Enter competing interest details beginning with this statement:<br><br>I have read the journal's policy and the authors of this manuscript have the following competing interests: [insert competing interests here]<br><br>* typeset	

Powered by Editorial Manager® and ProduXion Manager® from Aries Systems Corporation

Ethics Statement<br><br>Enter an ethics statement for this submission. This statement is required if the study involved:<br><br>• Human participants<br>• Human specimens or tissue<br>• Vertebrate animals or cephalopods<br>• Vertebrate embryos or tissues<br>• Field research<br><br>Write "N/A" if the submission does not require an ethics statement.<br><br>General guidance is provided below. Consult the submission guidelines for detailed instructions. Make sure that all information entered here is included in the Methods section of the manuscript.	This study was conducted in accordance with the Declaration of Helsinki and has been approved by the Ethics Committee of East China Normal University, with the approval number HR 695-2024.

Powered by Editorial Manager® and ProduXion Manager® from Aries Systems Corporation

Format for specific study types<br><br>Human Subject Research (involving human participants and/or tissue)<br>• Give the name of the institutional review board or ethics committee that approved the study<br>• Include the approval number and/or a statement indicating approval of this research<br>• Indicate the form of consent obtained (written/oral) or the reason that consent was not obtained (e.g. the data were analyzed anonymously)<br><br>Animal Research (involving vertebrate animals, embryos or tissues)<br>• Provide the name of the Institutional Animal Care and Use Committee (IACUC) or other relevant ethics board that reviewed the study protocol, and indicate whether they approved this research or granted a formal waiver of ethical approval<br>• Include an approval number if one was obtained<br>• If the study involved non-human primates, add additional details about animal welfare and steps taken to ameliorate suffering<br>• If anesthesia, euthanasia, or any kind of animal sacrifice is part of the study, include briefly which substances and/or methods were applied<br><br>Field Research<br><br>Include the following details if this study involves the collection of plant, animal, or other materials from a natural setting:<br>• Field permit number<br>• Name of the institution or relevant body that granted permission	
Data Availability<br><br>Authors are required to make all data underlying the findings described fully available, without restriction, and from the time of publication. PLOS allows rare exceptions to address legal and ethical concerns. See the PLOS Data Policy and FAQ for detailed information.	Yes - all data are fully available without restriction

Powered by Editorial Manager® and ProduXion Manager® from Aries Systems Corporation

A Data Availability Statement describing where the data can be found is required at submission. Your answers to this question constitute the Data Availability Statement and will be published in the article, if accepted.<br><br>Important: Stating ‘data available on request from the author’ is not sufficient. If your data are only available upon request, select ‘No’ for the first question and explain your exceptional situation in the text box.<br><br>Do the authors confirm that all data underlying the findings described in their manuscript are fully available without restriction?	

Powered by Editorial Manager® and ProduXion Manager® from Aries Systems Corporation

Describe where the data may be found in full sentences. If you are copying our sample text, replace any instances of XXX with the appropriate details.<br><br>• If the data are held or will be held in a public repository, include URLs, accession numbers or DOIs. If this information will only be available after acceptance, indicate this by ticking the box below. For example: All XXX files are available from the XXX database (accession number(s) XXX, XXX.).<br>• If the data are all contained within the manuscript and/or Supporting Information files, enter the following: All relevant data are within the manuscript and its Supporting Information files.<br>• If neither of these applies but you are able to provide details of access elsewhere, with or without limitations, please do so. For example:<br><br>Data cannot be shared publicly because of [XXX]. Data are available from the XXX Institutional Data Access / Ethics Committee (contact via XXX) for researchers who meet the criteria for access to confidential data.<br><br>The data underlying the results presented in the study are available from (include the name of the third party and contact information or URL).<br>• This text is appropriate if the data are owned by a third party and authors do not have permission to share the data.<br><br>* typeset	All relevant data are within the manuscript and its Supporting Information files.
Additional data availability information:	Tick here if your circumstances are not covered by the questions above and you need the journal's help to make your data available.

Powered by Editorial Manager® and ProduXion Manager® from Aries Systems Corporation

Manuscript

Click here to access/download;Manuscript;manuscript.docx

Abstract

The rise of artificial intelligence in the evolving world of information and communication technologies is reflected in profound educational changes. Higher education has been at the forefront of the changes, but vocational higher education does not seem to have received enough attention. Focusing on artificial intelligence literacy and sustainability of digital learning competences, this study examined the operational behaviors of 1004 students in a practical course in a higher education institution in mainland China by using the COM-B model. The results showed that AI literacy was positively correlated with sustainable digital learning ability, and AI literacy was positively correlated with students' sustainable digital learning behaviors through sustainability of digital learning competences. However, students in vocational institutions are not able to translate this learning behavior into a path to achieve good performance in practice, and even sustainability of digital learning competences can be slightly counterproductive. There is no excessive effect on the model after controlling for variables such as gender, family resources, and ethnic minorities, which contributes to SDG5 educational equity.

Keywords: AI literacy; Sustainable digital learning competences; SEM; Practice achievement; COM-B; vocational higher education

Commented [OS1]: The first sentence is a bit broad. You might clarify how AI's rise is specifically affecting education rather than stating it as a general trend.

Introduction

The pursuit of UN Sustainable Development Goal 4 (Quality Education) advocates for inclusive and equitable quality education for all [1] but there are gaps in its reality, gaps that both developed and developing countries. For instance, the proportion of the world's youth who are not in education, employment or training (NEET, Nyet) has increased from 21.8% in 2015-2019 to 23.3% in 2020, according to the UN Sustainable Development Goals Report 2022 [2]. This makes it necessary to focus on the cause of education and training among young people. In the field of education, the rise of Generative Artificial Intelligence (GAI) has directly brought about a dramatic change in digital transformation, which can provide transformative resolutions, but at the same time brings potential complexity to the field [3-7].

These changes may not only bring about a more efficient way to help teachers and students customize materials, but they also bring with them certain risks such as ethics [8]. For example, it may further widen inequities in education and disable some of the ability of students to learn. This inequity may be further increased for students in areas lacking ICT resources.

Not only that, there is also a gap between students' use of personal technology resources and their understanding of guidelines on the use of ICT for sustainable learning [9,10]. Therefore, it becomes important to properly utilize AI technology to find the right digital resources and transform them into sustainable learning.

More interestingly, there are essentially no rigorous rubrics to determine whether students are improving from AI use and the use of digital resources [11]. To enable

Commented [OS2]: Your discussion effectively highlights the challenges and opportunities AI presents in education, particularly in the context of SDG 4. However, some areas could be more concise and precise. The link between AI and inequities in education is well stated, but phrases like “disable some of the ability of students to learn” could be clearer—does this refer to over-reliance on AI or gaps in digital literacy? Additionally, the mention of ethics and inequity could be further connected to specific risks, such as algorithmic bias or accessibility issues. The argument about the lack of rigorous rubrics to assess AI’s impact is strong, but specifying what metrics or frameworks might be needed would add depth. The transition to China’s higher vocational education is relevant, but stating why this group is “not in the mainstream” would provide clarity. Overall, refining phrasing and strengthening logical connections between AI literacy, sustainable learning, and educational equity would improve the clarity and impact of the section.

better use of AI, data science education and data literacy may need to be integrated into K-12 education. As Chiu [12] suggests, the relationship between the two is currently not well-tested. In order to remedy these shortcomings and promote educational equity, the following issues are examined in terms of China's higher vocational education, a student who is not in the mainstream:

RQ1: Is there a correlation between AI literacy and sustainable digital learning competences?

RQ2: To what extent does AI literacy and the sustainable digital learning competences affect students' learning behaviors? Does this behavior facilitate or help students translate it into practical skills?

RQ3: Can AI literacy and sustainable digital learning competition contribute more to educational equity (SDG5) in a way?

Literature Review and Hypotheses Development

AI Literacy

The concept of AI literacy has been defined by a large number of scholars, and according to UNESCO [1], Ng [13], it can be defined in four parts: technology and impact, use and collaboration, evaluation and self-reflection, and ethics and morality. According to Ng's [13] account of consciousness and impact, 27 articles conceptualize AI literacy as educating learners to acquire fundamental concepts, skills, knowledge, and attitudes that do not require a priori knowledge. In addition to being the end user of an AI application, learners should also understand the technology behind it. Burgsteiner et al. [14] and Kandlhofer et al. [15] define AI literacy as the ability to understand the basic technologies and concepts behind AI in different products and services. In addition, some researchers have linked AI literacy to perceptual ability, confidence and readiness to learn AI. However, since we have not been able to find a way to apply this to vocational education, we can only blend this concept from K-12 education to higher education to get a more accurate explanation, for example, in the k12 section, Druga et al. [16] and Lin et al. [17] design learning lessons and activities to develop AI literacy, focusing on how learners acquire AI concepts. This section of the higher education perspective seems to go even deeper, requiring that teachers and students must understand the fundamentals of AI, such as machine learning at the foundational level, in order to critically assess when and how to incorporate AI tools into pedagogy, student assessment and evaluation, or to better facilitate the delivery of courses and learning [13,18,19].

Commented [OS3]: Your discussion provides a thorough overview of AI literacy, but some areas could be more concise and precise. The definition section is well-supported by literature, but phrases like *"educating learners to acquire fundamental concepts, skills, knowledge, and attitudes that do not require a priori knowledge"* could be clearer—does this mean AI literacy is accessible without prior expertise? The transition from K-12 to higher education is strong, but clarifying how vocational education specifically fits into this continuum would enhance clarity.

AI enhances human intelligence through digital automation, and 19 articles [13] mention that AI literacy can enable learners to participate in higher-order thinking activities and generate better interactions. In addition to understanding and using the concepts and practices of artificial intelligence, some studies have also extended AI literacy to two other abilities, enabling individuals to critically evaluate AI technology and effectively communicate and collaborate with AI [20]. For example, Han et al. [21] improved students' knowledge of science and technology, and then applied it to scientific research-based learning to solve practical problems. Long et al. [22] encourage citizens to participate in the co creation of AI facilities in public places to broaden their public AI literacy and experience. Participants can participate in the production of public interactive art works, from being initially attracted by AI devices to interacting with the devices and others. Overall, although these articles have slightly

different definitions of AI literacy, they all support the view that everyone, especially K-12 children, has acquired basic AI knowledge and abilities, enhanced motivation for future careers, and utilized AI technology [23]. In higher education, this interactivity is more evident, as AI technology is widely applied in the interaction between teachers and students, such as teaching and completing specific tasks [18,19,24,25]. For the assessment and self-reflection phase it seems that k-12 appears to be somewhat inadequate. Many people claim to know little about AI [20]. Nevertheless, “folk theories” (i.e., “informal theories ...... sense and explain how systems work") to explain AI algorithms [26]. These theories, whether accurate or not, shape the nature of user interaction and experience [26] which seems to be reduced to a previous stage of use and collaboration. A better understanding of how AI works can help people form more accurate mental models of the systems they interact with. Higher education has shown more interest in this area, and Dong [20] conducted a review of topics covered in university syllabi, including machine learning [27,28], AI [29] writing up a curriculum A list of all topics covered in the curriculum was written, as well as a list of learning objectives for the K-12 audience in the AI education program [16,30]. Topics range from high-level concepts (e.g., learning, kinematics, planning) to concrete implementations (e.g., Bayesian networks, Markov models). Most syllabi are aimed at computer science majors, and many courses require that K-12 programs also require students to have some prerequisite knowledge of mathematics, statistics, or computer science. This level of prerequisite knowledge may make these courses and their content inaccessible to groups that could benefit from AI literacy, such as children who interact with AI at home or adults who use AI in the workplace [31]. Considering the specificity of higher vocational institutions and the lack of pre-learning for students about AI courses, it is important for assessment and self-reflection to focus more on assessing the wider societal impact of AI, including its potential benefits and risks, and how it may affect education and future work.

Ethical Adoption UNESCO's [32] first global guidance on GenAI in education aims to support countries in taking immediate action, planning long-term policies, and developing human capacity to ensure human-centered guidance on these new technologies. The guidance assesses the potential risks that GenAI may pose to the promotion of core human values of human agency, inclusiveness, equity, gender equality, linguistic and cultural diversity, and plurality of views and expressions. It proposes key steps for government agencies to regulate the use of GenAI tools, including mandating data privacy protections and considering age limits for use. It outlines requirements for GenAI providers to ensure their ethical and effective use in education. The guide emphasizes the need for educational institutions to validate the ethical and pedagogical applicability of GenAI systems in education. It calls on the international community to reflect on its long-term impact on knowledge, teaching, learning, and assessment. The publication provides concrete recommendations for policymakers and educational institutions on how to design the use of GenAI tools to protect human subjectivity and truly benefit learners, teachers, and researchers. In summary, the questionnaire used for the AI literacy of students in higher vocational education addresses the four dimensions of influence, cooperation, self-reflection, and ethics, adapted from Ieva [18], UNESCO [32], etc., and balances the basic content from the k-12 [20,27-29]and the self-reflection from the perspective of higher education [18,19,24,25].

Sustainability of Digital Learning Competences

In policy documents, academic literature, and teaching, learning, and certification practices, digital competence (DC) is interpreted in various ways (such as digital literacy, digital competence, electronic literacy, electronic skills, electronic competence, computer literacy, and media literacy) [33]. The International Institute of Computer and Information Literacy (ICILS) defines digital literacy/competence as an individual's ability to use computers for investigation, creation, and communication, in order to effectively participate in activities at home, school, workplace, and community. In line with this, several organizations such as UNESCO and OECD [34] have repeatedly called for research into digital learning competencies. For example, UNESCO's Global Digital Literacy Framework report emphasizes the use of technology for learning as a key digital literacy skill [35]. The National Conference of State Legislatures (NCSL) has included "the ability to use digital tools safely and effectively for learning" in the concept of digital competence/literacy [36], and Brolpito [37] emphasized the importance of digital competence for lifelong online learning. 19 pandemic further emphasizes the need for students to acquire digital learning competencies so that they can cope with this rapidly changing world. However, current literature does not focus in depth on digital learning competencies. Learning competence refers to the way individuals (and organizations as groups of individuals) identify, assimilate, and use knowledge [38]. The idea is that organisms have the capacity to learn, but there is something that enables them to learn, and that "something" (which is actually a lot of things) varies from person to person [39]. It is generally recognized that the ability to learn consists of three elements: goal (motivation), will and ability (knowledge and practice) [40]. This means that a person's learning ability depends entirely on whether he has a clear goal, strong will and rich theoretical knowledge and practical experience. Based on this, Yang defines digital learning competence as a set of knowledge, skills, and attitudes that enable students to learn effectively using digital tools in a digital learning environment [41]. We adopt the above concepts and qualify them with the expectation that students will be able to use digital resources sustainably for a long period of time or with a high frequency while learning each skill. In order to define sustainability, using the psychological example of a 12-week habit [42], we identified students as having Sustainable digital learning competences (or sustainability of digital learning competences, SDLC) if they were able to use digital resources for more than 21 consecutive days.

Based on the above definitions, we combined Ličen [43] and Yang [41] scales for determining digital learning ability to adapt them to finalize our measurement of students. Sustainability digital learning competence is finally measured by four dimensions: data literacy, data skills, digital interaction, and technology integration which are composed of four dimensions.

Digital literacy is a critical and evolving competency that goes beyond the basic use of digital tools. It encompasses a range of skills, knowledge and attitudes that are essential for the effective integration of digital technologies into teaching and learning. These include proficiency in the use of digital tools, integrating digital simulations, and facilitating student-to-student interactions through digital platforms, which are becoming increasingly important in modern education [43,44]. The ongoing digital transformation of higher education further emphasizes the importance of digital literacy as an essential element of effective learning. Digital literacy is dynamic and must evolve as new technologies emerge. This evolution is essential to promote equity, inclusion

and lifelong learning and is in line with global frameworks such as DigCompEdu [45,46].

Digital skills are another important dimension that focuses on the technical skills required to effectively use various digital tools and platforms in teaching. These skills include using learning management systems, digital collaboration tools, and specialized software that supports online and blended learning environments. Teachers and students must not only be proficient in using digital tools to enhance student engagement and learning outcomes [43]. This will create a wealth of learning opportunities for both teachers and students, which aligns with a broader digital competency framework that emphasizes the importance of specific digital skills for effective teaching and learning [46,47].

Digital interaction focuses on the use of digital tools to facilitate interactive learning environments. Technology integration refers to the incorporation of digital tools and resources into instructional practices to enhance learning. These two dimensions are critical for creating meaningful connections between students, teachers and content. The ability to interact and collaborate using digital platforms is increasingly recognized as the foundation of digital competence, especially in higher education where student engagement is a key factor in academic success [48-50]. This is in line with a growing body of research that assesses the role of digital tools in creating dynamic learning environments that promote active learning and collaboration [51]. **Specificity of vocational institutions**

Vocational institutions are not part of the mainstream values in China. According to Jiang et al.'s [52] study of the student population in Chinese vocational institutions, students have a very low status among the public and parents do not want their children to pursue specialized studies such as vocational technology. Chen [53] is even more outspoken in stating that vocational education is a dead end in the eyes of Chinese parents. This shows that this group is at a disadvantage in Chinese culture. In addition, vocational education lacks curriculum standards [54], and it seems that AI literacy and digital learning skills simply do not get any representation here. , this group has almost become a disadvantaged group, so it is beneficial to conduct research here to promote the United Nations Sustainable Development Goals (SDGs), especially SDG 4 (Quality Education), SDG 5 (Gender Equality), SDG 9 (Industry, Innovation, and Infrastructure), and SDG 10 (Reducing Inequality) [55].

Commented [OS4]: Improve clarity, reducing redundancy, and reinforcing logical connections would strengthen this section.

COM-B Model

There are a number of models to consider in the study of sustainable digital learning capabilities and students' sustainable learning behaviors. For example, the Technology Acceptance Model (TAM) has also been used to study students' intentions towards AI. Yu [56] proposed a research model based primarily on the TAM to study students' behavioral intentions to use next-generation information technologies, including AI, in intelligent foreign language learning. As another example, Le Dang [57], in analyzing university students' intention to use AI for learning, used correlates from the Technology Acceptance Model (TAM), the Technology Readiness Model (TR), and the Technology Readiness and Acceptance Model (TRAM) to set behavioral intention as a dependent variable influenced by perceived usefulness and perceived ease of use. Chatterjee and Bhattacharjee [58] relied on a variety of theories and models including UTAUT to study the application of AI in higher education. They analyzed the effect of attitudes and facilitating conditions on behavioral intentions and the effect of behavioral intentions

Commented [OS5]: Your discussion effectively contrasts different theoretical models for studying AI adoption and sustainable digital learning behaviors, but it could be more concise.

on AI adoption. It seems that each model has a very relevant ability to take on this task, but all of these models have a rather interesting point: it is not possible to analyze all the independent variables by integrating them into one competency or one literacy overall consideration and behavior. Therefore, we hope to find a model that can solve such a trouble at a larger or more macro level.

COM-B aims to provide an overarching model that encompasses all the factors known to influence behavior change [59]. This model, proposed by the National Institute for Health and Care Excellence (NIHCE), cites the Capabilities, Opportunities, Motivations, Behaviors (COM-B) model [59] as a key theoretical framework for understanding and supporting behavior change [60].The COM-B model incorporates six elements that are believed to drive behavior, namely physical ability (having skills, strength and endurance); mental ability (being able to engage in necessary thought processes such as comprehension and reasoning); physical opportunity (provided by the environment, including time and resources); social opportunity (provided by interpersonal influences, social cues, and the way we think about things, such as the words and concepts that make up language); reflexive motivation (the ability to make a conscious intention, plan and making assessments); and automatic motivation (emotional reactions, impulses, and desires) [59,61]. This model is more macroscopic and allows for the three COMs to be examined separately as latent variables or merged together to collectively support as COM, which is very much in line with the indicator of digital learning competence for sustainability as this competence is an aggregate term as per the previous discussion, which prefers a holistic conceptualization of COM merged together [62].

Hypothesis

The Relationship between AI Literacy and Sustainability of Digital

Learning Competences

Before the emergence of artificial intelligence literacy, the term 'digital literacy' was used to evaluate basic concepts and skills related to computers, which began to be popular in various industries in the 1970s. Users must be proficient in using computer systems related to their specific tasks or work. As more and more people rely on computer technology to develop new social and economic opportunities, the importance of digital literacy is increasing. This concept gradually evolved into the digital literacy that follows [63].

For early digital literacy, this is a prerequisite for AI literacy, as individuals need to understand how to use computers to understand AI [20]. But as digital transformation becomes more and more successful, the concept of digital competence has been introduced, and new ways of connecting data, people and processes in modern digital conditions aim to create better environments and prepare for future challenges, in which case AI is one of the factors influencing the creation of a digital education infrastructure. For example Artificial Intelligence in vocational education can be observed in the practice of engineering students to be able to predict, monitor, optimize and plan the construction design of civil engineering works, which is similar in other engineering fields [64], which the students acquire during their professional training [65], and it is interesting to see that from the use of engineering students It is interesting to verify the technological progress, which is even more evident when they graduate and become

Commented [OS6]: Your discussion effectively traces the evolution from digital literacy to AI literacy, highlighting their interconnectedness. However, the transition between concepts could be more fluid, particularly in linking digital competence and AI's role in education. The engineering education example is relevant but could be more concisely integrated to strengthen the argument. The necessity of AI literacy in vocational education is well justified, but reducing redundancy would improve clarity. The hypothesis (H1) is logical and well-grounded in the preceding discussion.

professionals [66]. Likewise, it is important and necessary to include the development of technological tools in every course in the educational curriculum of higher education classrooms, as mentioned by González [67], the entire teaching staff will be consistent in the use of tools , prioritizing the training of each professor in the use of ICT [68,69], which indicates that the institution has its own regulations for the improvement of the quality of education or the content offered to students, which goes from the classroom presentations to the collaborative work carried out through the platforms, and especially in the assessment of each student [70,71] constant training in the use of different technologies. In summary, the hypotheca is made:

H1: AI literacy is beneficial for the improvement of sustainability of digital learning competences.

The Relationship between SDLC and Behavior

COM needs to be integrated with the previous SDLC, and based on the definition of COM, as well as the question set in the SDLC questionnaire and the previous discussion, it is argued here that data literacy is the competence, data skills are the opportunity, and digital interaction, and technology integration are the motivations.

The COM-B model represents the observation that at any given moment, a particular behavior will only occur if the person involved has the ability and opportunity to engage in that behavior and is more motivated to perform it than any other behavior. Sustainable digital learning behaviors are considered here as endemic behaviors. In order to measure this behavior, Ieva's [18] formulation of the behavior is used and combined with some of the statements made by Romero [72] in his study of AI usage behavior.

According to the COM-B model, we can make the following hypothesis:

H2: SDLC promotes students' digital learning behavior.

But given the externalized nature of the behavior, and in order to explore the validity of the behavior and make up for the lack of AI literacy and digital competence measurements, while, it seems that the use of AI and the use of digitization can make some tangible results, some form of externalization is still missing [21-23]. we're going to make a bolder guess:

H3: Students' digital learning behavior may promote their practical achievement.

Summarizing the previous arguments leads to the final hypothesis:

H4: AI literacy promotes students' digital learning behavior by enhancing the sustainability of digital learning competences.

H5: AI literacy promotes students' practical achievement by enhancing the sustainability of digital learning competences.

Farmework

Based on what has been discussed in the previous section, the questionnaire on AI literacy developed by Ieva [18], UNESCO [32], which consists of four sections: influence, collaboration, self-reflection, and ethics will be used to measure it. Sustainability of Digital Learning Competences (SDLC) will be measured using the Digital Learning Capability Questionnaire developed by Ličen [43] and Yang [41]. Ieva's [18] and Romero [72] will be used to determine students' digital learning behaviors and practice scores will be used to identify the externalized forms of such behaviors where SDLC-behavior is studied according to the COM-B model. The conceptual framework of this study is shown in Figure 1.

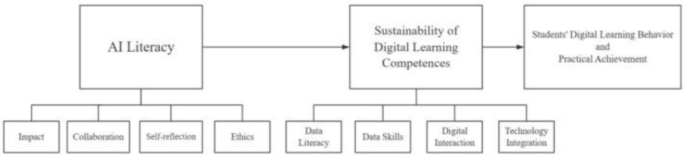

Fig1. Theoretical framework

Ethical Considerations

This study has obtained ethical approval from the Human Subject Research Ethics Committee of East China Normal University (Approval No. HR695-2024). All data is treated with confidentiality. All participants who are willing to participate in this study have obtained informed consent. According to the requirements of the ethics review committee, this article belongs to Class II exemption and does not involve sensitive information of the subjects. As the questionnaire is conducted online, an informed consent button is set in the questionnaire title. If you choose not to agree, the questionnaire will automatically end.

Methodology

Sample

A total of 1004 first year students (male = 869; female = 134) from four faculties of a higher vocational and technical school with a mean age of 19.93 years (SD = 2.645) participated in this study. The authors selected the study population using convenience sampling method with the help of local teachers. The students were predominantly Han Chinese, Manchu, Mongolian and Korean. The case school is a very famous vocational and technical school in Liaoning Province, and its teaching quality and students' academic performance are among the highest in the same industry in the region, and the employment rate of students is close to 100%. The average socio-economic status of the students' families is at the average level of Liaoning Province. Among them, the proportion of families with at least one person with a master's degree or above is 10.6%, the proportion of families with the highest education level of college or bachelor's degree is 21.7%, the proportion of families with the highest education level of senior high school or equivalent is 30.9%, and the proportion of families with the highest education level of junior high school is 36.9%. In particular, it should be noted that on the item of educational attainment, the sample is basically consistent with the real results of the Seventh National Population Census of the People's Republic of China, and the sample is highly representative [77]. All the specifics are shown in Table 1 below.

Table 1. Sample Description (N=1004)

Category	categories	Frequency	Percentage
Gender	Male	869	86.6%
	Female	134	13.4%

Age	16	121	12.1%
	17	121	12.1%
	18	112	11.2%
	19	113	11.3%
	20	106	10.6%
	21	109	10.9%
	22	90	9.1%
	23	105	10.5%
	24	126	12.5%
Parents' highest education level	middle school	370	36.9%
	high school or middle vocational school	310	30.9%
	high vocational school (associate degree) or bachelor	218	21.7%
	master or higher	105	10.6%
Minorities or Han	Han	678	67.6%
	Man	105	10.5%
	Mengguo	105	10.5%
	Chaoxian	115	11.5%
Family book collection	less than 10	204	20.8%
	10-50	361	36.2%
	50-100	178	16.7%
	over 100	261	26.3%

Questionnaire

This study used a questionnaire to investigate students' AI literacy and digital learning skills, and a five-point Likert scale (from “completely disagree” to “completely agree”) to assess students' level of agreement with these items. The questionnaire was ethically vetted to ensure that no personal privacy was involved. The questionnaire was designed through Questionnaire Star (https://www.wjx.cn/), which consisted of thirty-three questions, of which the ten questions in the first section were basic information about the individual, which contained some basic variables that needed to be controlled. Students' gender (male = 1; female = 2), age, ethnicity, parents' highest level of education (middle school = 1; high school or middle vocational school = 2; high vocational school(associate degree) or bachelor = 3; master's degree and above = 4), household resources and book ownership (i.e., how many books in the home), and number of books in the home) were included in the analyses as covariates, with book ownership, due to the relative prevalence of modern paperlessness, being the most common for the number of e-books was also included in the analysis as the number of books to control for its effect on motivation and achievement. Family resources can

represent the economic status of the family; book ownership was utilized for the measurement, not only that, but personal cell phones were not considered here, this is because filling out the questionnaire was disseminated using cell phone WeChat groups, which were filled out using the students' personal cell phones.

Artificial Intelligence Literacy

Artificial intelligence literacy was the second part of the questionnaire. This questionnaire was adapted from assessment of AI literacy [18-20,24,32]. The teacher section was removed and only the student section was studied. Due to the large number of questions, we filtered the questionnaire by selecting only the three questions with the highest reliability for each dimension. The questionnaire was divided into four dimensions as follows, Impact, Ethics, Collaboration and Self-reflection, the details of which are shown in Table 2 below. Not only that, but these four dimensions measure the dimension of AI literacy very well.

Table2. Reliability Analysis of AI Literacy Scale

Variables	Cronbach's alpha	Items
Impact (IM)	0.754	3
Ethics (ET)	0.824	3
Collaboration (CO)	0.840	3
Self-reflection (SR)	0.742	3
**AI Literacy**	**0.940**	**12**

The validation factor analysis was designed to test the validity of the four-factor structure of AI literacy values, $\square^2(df)\ \square\ 94.670(48)$, $p\square^{.001}$, $\square^2/df\ \square 1.972\ \square\ 3$, $RMSEA\square.031$, $GFI\ \square.985$, $RFI\ \square.983$, $CFI\ \square.994$, $IFI\ \square.994$, $TLI\ \square.992$ and indicate an acceptable model fit. Its structural validity is shown in Table 3 below.

Table3. Validity Testing of Differences in Various Dimensions of AI Literacy Scale

Variables	IM	ET	CO	SR
IM	**0.528**			
ET	0.391	**0.609**		
CO	0.397	0.669	**0.636**	
SR	0.407	0.68	0.684	**0.511**
Sqrt of AVE	**0.727**	**0.78**	**0.797**	**0.715**

Sustainability of Digital Learning Competences (SDLC)

Sustainable digital learning competences (SDLC) were the third part of the questionnaire. The questionnaire was adapted from Ličen and Prosen's assessment of teachers' competencies for sustainable digital learning [43] and Yang's [41] digital learning competencies questionnaire for students in 2024. The questionnaire is similar to the questionnaire for AI literacy, which was assessed for teachers, so adaptations were made to the teacher section to make it suitable for student research. Due to the large number of questions, we filtered the questionnaire and selected only the three questions with the highest reliability on each dimension. The questionnaire was categorized into the following four dimensions; Digital Literacy, Digital Skills, Digital Interaction, and Technology Integration, which are shown in Table 4. Not only that, but

these four dimensions also measure well the dimension of sustainable digital learning competences.

Table4. Reliability Analysis of Digital Learning Scale

	**Cronbach's alpha**	**Items**
Digital Literacy (DL)	0.819	3
Digital Skills (DS)	0.759	3
Digital Interaction (DI)	0.832	3
Technology Integration (TI)	0.865	3
**Digital Learning**	**0.949**	**12**

The validation factor analysis was designed to test the validity of the four-factor structure of digital learning values, □²(*df*) □ 57.770(48) , *p*□.001 , □² / *df* □1.204 □ 3 , *RMSEA*□.014 , *GFI* □.990 , *RFI* □.991 , *CFI* □.999 , *IFI* □.999 , *TLI* □.998 and indicate an acceptable model fit. Its structural validity is shown in Table 5 below.

Table5. Validity Testing of Differences in Various Dimensions of Digital Learning Scale

Variables	DL	DS	DI	TI
DL	**0.602**			
DS	0.678	**0.529**		
DI	0.691	0.724	**0.622**	
TI	0.688	0.713	0.712	**0.684**
Sqrt of AVE	**0.776**	**0.727**	**0.789**	**0.827**

Practice Achievement and Control Variables

In the spring of 2024, students received training from three teachers on the use of AI and digital tools for learning. The training consisted of four courses, and after class, students were required to use AI or digital tools for weekly learning and searching for materials to assist and guide students in practical operations. This course indirectly lasts for one semester, and students are initially competent in the use of AI and digital resources, and according to the 12-week nurturing principle [42], they can be considered to have sustainable digital learning competencies.

In order to evaluate practice performance more objectively, this section is divided into two areas. The first aspect is the operation results given by the school's professional teachers. The results are given by three expert teachers with deputy senior title, using 100 points and 60 points through the judging mode, and have more stringent judging standards, because the talents trained in this school are mainly for the service of the heavy industry. Many of these operations are dangerous, and if the operation is not done properly, it may lead to personal safety cannot be fully guaranteed, so this judgment will not be passed if the teacher determines that it is dangerous. Therefore, if the teacher determines that the operation is dangerous, the course will not be passed, so the failure rate of this course is high. Among them, 323 students failed this test (32.17%), and the remaining 681 passed the test (67.83%), and among the groups that passed the test, the group with a score of 60 to 79 was 327 (32.57%), and the group with a score of 80 to 100 was 354 (35.26%); it is very interesting to note that each of the three groups of failing and good and good each accounted for more than one-third. A reasonable

explanation for this is that since there are three teachers grading, if two of them give a low score, they will get a direct fail; if two of them give a pass and one of them gives a fail, they will get a good score; and if all three of them give a pass, they will get a high score.

The second part was derived from the students' self-assessment or behavior, and the test consisted of three questions that were adapted from Ieva and Daniela [18] for the measurement of the use of AI and digital behaviors (3items,). Their specific information is set out below:

1. I am aware that there are different AI tools and digital resources that can be used in different educational courses (e.g. ChatGPT, Tiangong, Doubao, Learning Power, MOOC).
2. I have tried at least once a week to use AI tools and digital resources to plan or participate in the learning process to support my learning process (e.g., preparing lesson ideas, creating presentations, fact-checking).
3. I use AI tools and digital resources to support the learning process and find a variety of AI tools and a variety of digital resources based on different learning needs and abilities, with a basic understanding of their potential mechanisms (e.g., providing textto-speech tools, using AI tools to help check answers, finding learning courses that can support manipulation, etc.).

These three topics appeared to have a Cronbach coefficient that was not very high, and in order to understand the deeper reasons why this arose, we did a short, small-scale informal interview, and a plausible explanation is that many students were aware of the possibility of using these techniques and applications to facilitate their practical learning, but it was difficult to find these practical exercises on the web, which made the topics less reliable. After discussion between several researchers, we unanimously decided to still use these data, this is because the content of this questionnaire has been developed by other researchers [18] and has achieved relatively good reliability and there is literature to support this claim [73,74].

Data Analysis

The questionnaires were pre-set to exclude those that were randomly filled out, and those that took less than 100 seconds to answer, or that had the same value for all questions, were simply deleted. In addition to this, no missing values will occur as each question has a mandatory answer requirement.

First, we utilized SPSS to perform descriptive statistics for the questionnaire. Second, we used Structural Equation Modelling (SEM) and maximum likelihood estimation of AMOS to examine the relationship between AI Literacy, Digital Learning and practice achievement. In this study, $\square^2 / df \square 3$, $RMSEA \square .08$ $GFI \square .90$, $RFI \square .90$, $CFI \square .90$, $TLI \square .90$

[78] indicated an acceptable model fit. Third, we will utilize AMOS to investigate path salience and some further exploration.

Results

The conclusions of this paper are divided into four parts; the first part examines the SEM model between AI literacy and digital learning; the second part examines the SEM model of AI and digital learning and student behavior; the third part examines the model of AI literacy and student achievement in practice; and the fourth part concludes the multi-cluster analysis.

Structural Equation Model 1

In Model 1, two variables, AI literacy and sustainable digital learning competences, were included in the study. The SEM results showed that our hypothesized model fitted the data well: □^{2}(*df*) □ 375.106(251) , *p*□$^{.001}$, □^{2}/ *df* □1.494 □ 3 , *RMSEA*□.022 , *GFI* □.969 , *RFI* □.978,*CFI* □.993, *IFI* □.993,*TLI* □.993.

Our results predict a very strong positive correlation between AI literacy and sustainable digital learning competences (□□.998, *p*□.001). In the section on AI literacy, each observable variable has a relatively good explanation of AI literacy. For the impact section, each of the three topics explained AI literacy positively (□$_1$ □.554, *p*□.001 , □$_2$ □.786, *p*□.001,□$_3$ □.806, *p*□.001). For the ethics section, each of the three topics was positively linked to an explanation of AI literacy (□$_1$ □.785, *p*□.001 ,□$_2$ □.789, *p*□.001 , □$_3$ □.779, *p*□.001). For the collaboration section, each of the three topics was positively linked to an explanation of AI literacy (□$_1$ □.780, *p*□.001 , □$_2$ □.786, *p*□.001 , □$_3$ □.822, *p*□.001). For the self-reflection section, each of the three topics was positively associated with an explanation of AI literacy (□$_1$ □.795, *p*□.001 , □$_2$ □.510, *p*□.001 , □$_3$ □.788, *p*□.001).

In the section Sustainable digital learning competences, each observable variable can be reasonably explained. For the digital literacy component, each of the observed variables was positively associated with SDLC (□$_1$ □.781, *p*□.001 , □$_2$ □.779, *p*□.001 , □$_3$ □.777, *p*□.001). For the digital skills section, each of the three topics was positively linked to an explanation of SDLC (□$_1$ □.800, *p*□.001,□$_2$ □.543, *p*□.001,□$_3$ □.798, *p*□.001).

For the digital interaction section, each of the three topics was positively associated with an explanation of SDLC (□$_1$ □.800, *p*□.001,□$_2$ □.787, *p*□.001,□$_3$ □.782, *p*□.001). For the technology integration section, each of the three topics was positively linked to an explanation of SDLC (□$_1$ □.787, *p*□.001,□$_2$ □.889, *p*□.001,□$_3$ □.801, *p*□.001).

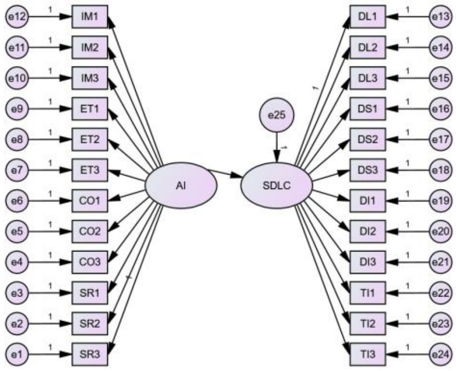

Fig2. SEM of AI Literacy and Sustainable Digital Learning Competences

Structural Equation Model 2

In Model 2, AI literacy and sustainable digital learning competencies and student behaviors were included in the study. The SEM results showed that our hypothesized model fitted the data well:□^{2}(*df*) □ 529.050(322) , *p*□$^{.001}$,□^{2}/ *df* □1.643 □ 3, *RMSEA*□.025 , *GFI* □.961, *RFI* □.971,*CFI* □.990, *IFI* □.990,*TLI* □.989.

In this model, the positive correlation between AI literacy and sustainable digital learning competencies was further corroborated (□□.999, *p*□.001), and achievable digital learning competencies also produced a significant positive correlation with student behavior (□□.825, *p*□.001).

In the section on AI literacy, each observable variable has a relatively good explanation of AI literacy. For the impact section, each of the three topics explained AI literacy positively (□$_1$ □.558, *p*□.001,□$_2$ □.786, *p*□.001,□$_3$ □.806, *p*□.001). For the ethics section, each of the three topics was positively linked to an explanation of AI literacy (□$_1$ □.785, *p*□.001,□$_2$ □.789, *p*□.001,□$_3$ □.778, *p*□.001). For the collaboration section, each of the three topics was positively linked to an explanation of AI literacy (□$_1$ □.779, *p*□.001 , □$_2$ □.785, *p*□.001 , □$_3$ □.822, *p*□.001). For the self-reflection section, each of the three topics was positively associated with an explanation of AI literacy (□$_1$ □.795, *p*□.001,□$_2$ □.514, *p*□.001,□$_3$ □.788, *p*□.001). In the section Sustainable digital learning competences, each observable variable can be reasonably explained. For the digital literacy component, each of the observed variables was positively associated with SDLC (□$_1$ □.780, *p*□.001,□$_2$ □.780, *p*□.001,□$_3$ □.776, *p*□.001). For the digital skills section, each of the three topics was positively linked to an explanation of SDLC (□$_1$ □.798, *p*□.001,□$_2$ □.547, *p*□.001,□$_3$ □.797, *p*□.001). For the digital interaction section, each of the three topics was positively associated with an explanation of SDLC (□$_1$ □.800, *p*□.001 , □$_2$ □.787, *p*□.001 , □$_3$ □.783, *p*□.001). For the technology integration section, each of the three topics was positively linked to an explanation of SDLC (□$_1$ □.787, *p*□.001,□$_2$ □.888, *p*□.001,□$_3$ □.801, *p*□.001). It can be seen that in contrast to

Model 1, the standardized estimates of each observable variable are more stable and reflect the latent variables well. For student behavior, the three observables provide good positive feedback (□$_1$ □.633, *p*□.001,□$_2$ □.614, *p*□.001,□$_3$ □.675, *p*□.001). The significant paths are shown by Figure 3 below.

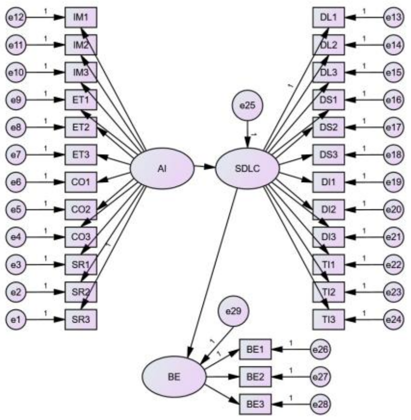

Fig3. SEM of AI Literacy, Sustainable Digital Learning Competencies and Behavior

Structural Equation Model 3

In Model 3, AI literacy and sustainable digital learning competencies and practice achievement were included in the study. The SEM results showed that our hypothesized model fitted the data well:□^{2}(*df*) □ 395.781(273) , *p*□$^{.001}$,□^{2}/ *df* □1.450 □ 3, *RMSEA*□.021,

GFI □.969 , *RFI* □.977,*CFI* □.993, *IFI* □.993,*TLI* □.993. In this model, there is a positive correlation between AI literacy and sustainable digital learning competencies (□□.998, *p*□.001), and a positive correlation with practice achievement

(□□.390, *p*□.904). Digital learning competencies showed a negative correlation with practice grades (□□□.394, *p*□.903). Unfortunately, however, the paths to practice achievement were not significant for either AI literacy or SDLC. It can be assumed that student selves have been using AI-type technology tools to design and learn much of the content, but from a practical standpoint, such help or feedback may have had minimal effect.

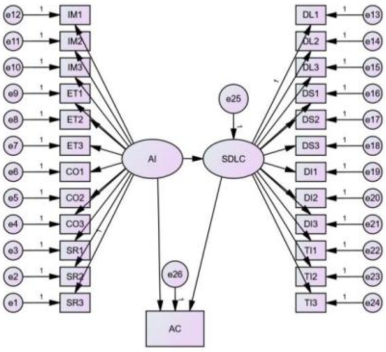

Fig4. SEM of AI Literacy, SDLC and Practice Achievement

Control Variables Analysis

A multi-cluster analysis was performed for Model 2.

Gender

For gender, the model is stable. The fit is good for unconstrained models:

□²(*df*) □ 915.774(644) , *p*□.001 , □²/ *df* □1.422 □ 3 , *RMSEA*□.021 , *RFI* □.951 , *CFI* □.986 , *IFI* □.986,*TLI* □.985. Not only that, the unconstrained model passes the hypothesis test (measurement weights □²(*df*) □ 23.798(24) , *p*□.473). A similar situation occurs with measurement weights (□²(*df*) □ 939.572(688) , *p*□.001 , □²/ *df* □1.407 □ 3 , *RMSEA*□.020 , *RFI* □.952 , *CFI* □.986 , *IFI* □.986 , *TLI* □.986) and with structural weights (□²(*df*) □ 941.168(670) , *p*□.001 , □²/ *df* □1.404 □ 3 , *RMSEA*□.020 , *RFI* □.952 , *CFI* □.986 , *IFI* □.986,*TLI* □.986).

Parents' highest education level

The highest level of parental education also appeared to be insensitive to the model created. The fit is good for unconstrained models: □²(*df*) □1722.556(1400) , *p*□.001 ,

□²/ *df* □1.230 □ 3, *RMSEA*□.015 , *RFI* □.918,*CFI* □.984, *IFI* □.984,*TLI* □.984. Not only that, the unconstrained model passes the hypothesis test (measurement weights □²(*df*) □18.462(24) , *p*□.780). A similar situation occurs with measurement weights

(□²(*df*) □1741.018(1424) , *p*□.001 , □²/ *df* □1.223 □ 3 , *RMSEA*□.015 , *RFI* □.919 , *CFI* □.984 , *IFI* □.984 , *TLI* □.984) and with structural weights (□²(*df*) □1724.178(1426) , *p*□.001 , □²/ *df* □1.222 □ 3, *RMSEA*□.015 , *RFI* □.919,*CFI* □.984, *IFI* □.984,*TLI* □.984).

Minorities or Han

Minority or Han Chinese had no effect on the unconstrained model, but had a slight effect on the path. The fit is good for unconstrained models: □²(*df*) □1843.115(1400) , *p*□.001 ,□²/ *df* □1.317 □ 3 , *RMSEA*□.018 , *RFI* □.914 ,*CFI* □.978 , *IFI* □.978 ,*TLI* □.978 . The biggest difference comes from the Manchus, whose path is somewhat different from the other three ethnic groups. For example, the estimates on SDLC to student behavior (□□.819 , *p*□.001) are slightly lower than the other three ethnic groups (□□.828 , *p*□.001).

It can be seen that the model 2 is not sensitive to several clusters, which is a greater indication of the inclusiveness and fairness of AI literacy and Sustainable digital learning competences.

Discussion

Overall, both the AI literacy questionnaire and the SDLC questionnaire had good reliability and validity, and the four sub-dimensions were able to clearly measure the whole question. Several structural equation models fit well and explained the previous hypotheses completely.

Commented [OS7]: The statement effectively conveys the reliability and validity of the questionnaires, as well as the strong model fit. However, it could briefly mention any key implications or limitations to provide a more comprehensive conclusion.

AI Literacy and Sustainable Digital Learning Competencies

The results showed a strong positive correlation between AI literacy and sustainability digital learning ability, an observation that is consistent with previous research [20]. From an impact perspective, this concept is highly volatile for the measurement of AI literacy because the questions are more conceptually oriented, an issue that cannot be avoided in either K-12 [16,17], or higher education contexts [13,18,19]. Although we preferred a k-12 setting in this section that focuses on learners acquiring the concept of AI, considering that the level of the students is not sufficient to support the condition of having a high level of math and basic prior knowledge, in terms of impact despite the greater success, there is still room for upward mobility that can enable students to look at the concept of AI in terms of discarding a part of their foundation in math. This coincides with some previous research on how to design questions to critically assess

when and how to incorporate AI tools into pedagogy, student assessment and evaluation, or to better facilitate the delivery of courses and learning [13,18,19].
A similar situation to the AI impact aspect occurs in the self-reflection section, where large swings occur. This is more related to the K-12 section for this issue, for example Long [20] is outspoken about how little is known about this section, some folk theories to establish algorithms for AI which, whether accurate or not, shape the nature of user interactions and experiences [26], which seems to be reduced to the previous stage of use and collaboration. This issue has been transformed in the context of higher education, but given the level of students, this level of prerequisite knowledge may make these courses and their content inaccessible to groups that may benefit from AI literacy [31], so the question is being designed to think more about the broader societal implications of AI, including its potential benefits and risks, and how it may affect education and the future work, which brings the question back to the k-12 level when targeting students in higher vocational institutions, ultimately leading to some volatility. This situation does not seem to exist in terms of ethics and morals, probably thanks to the fact that the content used is adapted from UNESCO [32], which makes the title free of such problems due to the large sample size and the authority of the experts. Collaboration also has a very good explanation for AI literacy, which is highly similar to many previous studies [20-22]. As Chai [23] states: everyone, especially K-12 children, have gained basic AI knowledge and competence, enhanced motivation for future careers, and utilized AI technology.

Sustainable Digital Learning Competencies and Behavior

In Model 2, SDLC has a very strong and positive effect on students' digital learning behavior. Not only that, there is a further increase in the positive correlation between AI literacy and SDLC.
The possible reason for the fluctuation in the interpretation of data skills for SDLC is that such a requirement may be too high for students in vocational institutions. As stated in the previous definition, data skills include the use of learning management systems, digital collaboration tools, and specialized software that support online and blended learning environments. Teachers and students must not only be proficient in using digital tools to improve student engagement and learning outcomes [43]. This may be too high an expectation for these academically disadvantaged groups [53].
One possible explanation for digital literacy being relatively stable for SDLC measurement is that a range of skills, knowledge and attitudes are covered in the measurement process, and these factors may have been somewhat relaxed relative to the rigid requirements for digital skills. This is also in line with the fact that digital literacy is dynamic, and that this development is conducive to for the promotion of equity, inclusion, and lifelong learning [45, 46].
The two dimensions of digital interaction and technology integration also showed notable results, and one possible explanation is that the use of digital tools to facilitate interactive learning environments and thus increase student engagement was generally recognized by students, which is consistent with previous research [48-51].
A more unfortunate point is that the data does not support H4. Students' practical performance is not strongly linked to AI literacy (i.e., the ability to learn digital technologies in a sustainable way). This may be related to the following two reasons. Firstly, it is not clearly documented in the existing literature that one's practical competence on a given problem can be significantly guided by the use of AI or the use of data resources, e.g., as mentioned earlier, AI can be observed in the practice of

engineering students enabling them to predict, monitor, optimize, and plan the construction design of a civil engineering project [64], but it does not mean that the that the competencies acquired by the students can be well measured [11]. Secondly, it was also learned through informal interviews with the students that although the students were more enthusiastic about searching for information packages on the Internet, there was a gap between the online information and the practice, and the lack of practical content and videos did not allow the students to understand the practical content very well. Again, the disadvantage of vocational college students, some students have a reverse psychology to digital learning, as Jiang [52] said, Chinese parents are not willing to let students go to technical schools, resulting in the group of technical school students become a disadvantaged group in China's competitive system, the students will be passive and unable to transform the knowledge they have learned into external capabilities. It is worth noting that practice class grades show a negative effect with having a higher SDLC, and while this pathway is not significant, some effects do appear. One possible explanation is that students are tired of such a model of developing digital learning literacy and the negative effect occurs because of internal resistance. This explanation can be corroborated with some of Chen's [53] claims that neither parent expects anything from these children because they are not happy to do anything.

Control Variables for Model Impact

The article examined three control variables, namely gender, parents' highest level of education, and ethnic minority versus Han Chinese.

In terms of gender, the model is very stable, and this stability is not only for the unconstrained model, but also for the weights, indicating that the model is well fitted and that gender does not play a large role in this model. This is a very good sign that the impact of AI literacy and sustainable digital competence on student behavior is not interfered by gender factors. This validates some previous research [45,46] that AI and digital resources greatly contribute to gender equity, which is very much in line with SDG5 [55,76]. It also provides new ideas for vocational and adult education, utilizing AI and digital resources without gender discrimination [75,76].

Not only that, this model for the parents of the highest education, the above mentioned ethnic or Han also reflects a surprisingly inclusive, regardless of the level of parental education, regardless of the family's economic situation, regardless of ethnic minorities or not, can enjoy the AI and digital resources to bring more fair, enjoy equal and quality education resources [55].

Conclusions

This study examined the relationship between AI literacy, sustainable digital learning competencies, students' digital learning behaviors and practical performance with 1004 senior students in Liaoning Province. The results of the study confirm that AI literacy can enhance sustainable digital learning ability, and AI literacy positively affects students' digital learning behaviors through sustainable digital learning ability, but it does not have a strong effect on practice grades. This somewhat supports some previous studies [13, 18-23]. In addition, this paper finds that the use of AI literacy and digital resources can contribute more to the equity of education, which includes not only gender, but also family economic conditions, ethnic minorities or Han Chinese, which fits well with the United Nations SDG 4, SDG5.

References

1. UNESCO. Guidance for Generative AI in Education and Research. Available online: https://www.unesco.org/en/articles/guidance-generative-ai-education-andresearch (accessed on 28 June 2024).
2. UN. The Sustainable Development Goals Progress Chart 2022. Available online: https://unstats.un.org/sdgs/report/2022/
3. Lai, C.Y.; Cheung, K.Y.; Chan, C.S. Exploring the role of intrinsic motivation in ChatGPT adoption to support active learning: An extension of the technology acceptance model. Comput. Educ. Artif. Intell. 2023, 5, 100178.
4. Al Yakin, A.M.; Obaid, A.J.; Abdul, L.; Warsah, I.; Muthmainnah, M.; Elngar, A.A. Harnessing the Power of ChatGPT to Explore Student Metacognitive Skills in Learning Sociology Education. In Advanced Applications of Generative AI and Natural Language Processing Models; Obaid, A., Bhushan, M.S., Rajest, S., Eds.; IGI Global: Hershey, PA, USA, 2024; pp. 405–423.
5. Raman, R.; Pattnaik, D.; Hughes, L.; Nedungadi, P. Unveiling the dynamics of AI applications: A review of reviews using scientometrics and BERTopic modeling. J. Innov. Knowl. 2024, 9, 100517.
6. Linda, S. A State-of-the-Art Review on the Implications of ChatGPT in Educational Practices and Research. In Educational Perspectives on Digital Technologies in Modeling and Management; Prakasha, G., Lapina, M., Balakrishnan, D., Sajid, M., Eds.; IGI Global: Hershey, PA, USA, 2024; pp. 1–24
7. Jeon, J.; Lee, S. Large language models in Education: A focus on the complementary relationship between human teachers and ChatGPT. Educ. Inf. Technol. 2023, 28, 15873–15892.
8. Holmes, W.; Porayska-Pomsta, K.; Holstein, K.; Sutherland, E.; Baker, T.; Shum, S.B.; Santos, O.C.; Rodrigo, M.T.; Cukurova, M.;Bittencourt, I.I. Ethics of AI in education: Towards a community-wide framework. Int. J. Artif. Intell. Educ. 2022, 32, 504–526.
9. Sayaf, A.M.; Alamri, M.M.; Alqahtani, M.A.; Al-Rahmi, W.M. Information and Communications Technology Used in Higher Education: An Empirical Study on Digital Learning as Sustainability. Sustainability 2021, 13, 7074.
10. Zermeño Flores, A.I.; González Sánchez, R.F.; Cruz García, L.E. TIC en la calidad de vida de jóvenes universitarios: Un modelo integral desde el enfoque de capacidades. Disert. Anu. Electrónico Estud. Comun. Soc. 2024, 17, 3.
11. Lorena Casal Otero; Catala, A.; Fernández-Morante, C.; Taboada, M.; Beatriz Cebreiro López; Barro, S. AI Literacy in K-12: A Systematic Literature Review. International Journal of STEM Education 2023, 10 (1). https://doi.org/10.1186/s40594-023-00418-7.
12. Chiu, T. K. F.; Ahmad, Z.; Ismailov, M.; Sanusi, I. T. What Are Artificial Intelligence Literacy and Competency? A Comprehensive Framework to Support Them. Computers and Education Open 2024, 6, 100171. https://doi.org/10.1016/j.caeo.2024.100171.
13. Ng, D. T. K.; Leung, J. K. L.; Chu, S. K. W.; Shen, M. Q. Conceptualizing AI Literacy: An Exploratory Review. Computers and Education: Artificial Intelligence 2021, 2 (1), 100041. https://doi.org/10.1016/j.caeai.2021.100041.

Commented [OS8]: References are adequate.

14. Burgsteiner, H.; Kandlhofer, M.; Steinbauer, G. IRobot: Teaching the Basics of Artificial Intelligence in High Schools. Proceedings of the AAAI Conference on Artificial Intelligence 2016, 30 (1). https://doi.org/10.1609/aaai.v30i1.9864.
15. Kandlhofer, M.; Steinbauer, G.; Hirschmugl-Gaisch, S.; Huber, P. Artificial Intelligence and Computer Science in Education: From Kindergarten to University. 2016 IEEE Frontiers in Education Conference (FIE) 2016. https://doi.org/10.1109/fie.2016.7757570.
16. Druga, S.; Vu, S. T.; Likhith, E.; Qiu, T. Inclusive AI Literacy for Kids around the World. Proceedings of FabLearn 2019 2019. https://doi.org/10.1145/3311890.3311904.
17. Lin, P.-Y.; Chai, C.-S.; Jong, M. S.-Y.; Dai, Y.; Guo, Y.; Qin, J. Modeling the Structural Relationship among Primary Students' Motivation to Learn Artificial Intelligence. Computers and Education: Artificial Intelligence 2021, 2, 100006. https://doi.org/10.1016/j.caeai.2020.100006.
18. Ieva Tenberga; Daniela, L. Artificial Intelligence Literacy Competencies for Teachers through Self-Assessment Tools. *Sustainability* 2024, *16* (23), 10386–10386. https://doi.org/10.3390/su162310386.
19. Wang, B.; Rau, P.-L. P.; Yuan, T. Measuring User Competence in Using Artificial Intelligence: Validity and Reliability of Artificial Intelligence Literacy Scale. Behaviour & Information Technology 2022, 42 (9), 1–14. https://doi.org/10.1080/0144929x.2022.2072768.
20. Long, D.; Magerko, B. What Is AI Literacy? Competencies and Design Considerations. Proceedings of the 2020 CHI Conference on Human Factors in Computing Systems 2020, 1–16. https://doi.org/10.1145/3313831.3376727.
21. Han, X.; Hu, F.; Xiong, G.; Liu, X.; Gong, X.; Niu, X.; Shi, W.; Wang, X. Design of AI + Curriculum for Primary and Secondary Schools in Qingdao. 2018 Chinese Automation Congress (CAC) 2018. https://doi.org/10.1109/cac.2018.8623310.
22. Long, D.; Jacob, M.; Magerko, B. Designing Co-Creative AI for Public Spaces. Proceedings of the 2019 on Creativity and Cognition 2019. https://doi.org/10.1145/3325480.3325504.
23. Chai, C. S.; Lin, P.-Y.; Jong, M. S.; Dai, Y.; Chiu, T. K. F.; Huang, B. Factors Influencing Students' Behavioral Intention to Continue Artificial Intelligence Learning. IEEE Xplore, 2020. https://doi.org/10.1109/ISET49818.2020.00040.
24. Sharples, M. Towards Social Generative AI for Education: Theory, Practices and Ethics. Learning: Research and Practice 2023, 9 (2), 159–167. https://doi.org/10.1080/23735082.2023.2261131.
25. Labadze, L.; Grigolia, M.; Machaidze, L. Role of AI Chatbots in Education: Systematic Literature Review. International Journal of Educational Technology in Higher Education 2023, 20 (1). https://doi.org/10.1186/s41239-023-00426-1.
26. Eslami, M.; Vaccaro, K.; Lee, M. K.; Elazari Bar On, A.; Gilbert, E.; Karahalios, K. User Attitudes towards Algorithmic Opacity and Transparency in Online Reviewing Platforms. Proceedings of the 2019 CHI Conference on Human Factors in Computing Systems 2019. https://doi.org/10.1145/3290605.3300724.
27. MacLennan, B. Introduction to Machine Learning. Kybernetes 2010, 39 (8). https://doi.org/10.1108/k.2010.06739hae.001.
28. Ng, A. Machine Learning | Coursera. Coursera. https://www.coursera.org/learn/machine-learning.
29. Charikar, M. Index of /class/cs221. Stanford.edu. http://web.stanford.edu/class/cs221/ (accessed 2025-01-05).

30. Touretzky, D.; Gardner-McCune, C.; Martin, F.; Seehorn, D. Envisioning AI for K-12: What Should Every Child Know about AI? Proceedings of the AAAI Conference on Artificial Intelligence 2019, 33 (01), 9795–9799. https://doi.org/10.1609/aaai.v33i01.33019795
31. Shapiro, R. B.; Fiebrink, R.; Norvig, P. How Machine Learning Impacts the Undergraduate Computing Curriculum. Communications of the ACM 2018, 61 (11), 27–29. https://doi.org/10.1145/3277567.
32. Miao, F.; Holmes, W. Guidance for Generative AI in Education and Research; UNESCO, 2023. https://doi.org/10.54675/ewzm9535.
33. Gallardo-Echenique, E.E.; Marqués-Molias, L.; de Oliveira, J.M. Digital Competence in the Knowledge Society. MERLOT J. Online Learn. Teach. 2015, 11, 1–16.
34. OECD. Students, Computers and Learning: Making the Connection, PISA; OECD Publishing: Paris, France, 2015.
35. Law, N.; Woo, D.; de la Torre, J.; Wong, G. A Global Framework of Reference on Digital Literacy Skills for Indicator 4.4.2; UNESCO Institute Statistics: Montreal, QC, Canada, 2018.
36. National Conference of State Legislature (NCSL). Digital Literacy. Washington, DC: The NCSL Podcast. NCSL. Available online: http://www.ncsl.org/research/education/digital-literacy.aspx (accessed on 11 April 2021).
37. Brolpito, A. Digital Skills and Competence, and Digital and Online Learning; European Training Foundation: Turin, Italy, 2018.
38. Van Winkelen, C.; McKenzie, J. Knowledge Works: The Handbook of Practical Ways to Identify and Solve Common Organizational Problems for Better Performance; John Wiley & Sons: New York, NY, USA, 2011.
39. Mercado, E. Mapping Individual Variations in Learning Capacity. Int. J. Comp. Psychol. 2011, 24, 4–35. https://doi.org/10.1016/j.jebdp.2005.01.011
40. Learning Capacity. Available online: http://wiki.mbalib.com/wiki/%E5%AD%A6%E4%B9%A0%E5%8A%9B (accessed on 25 February 2021).
41. Yang, J.; Tlili, A.; Huang, R.; Zhuang, R.; Bhagat, K.K. Development and Validation of a Digital Learning Competence Scale: A Comprehensive Review. Sustainability 2021, 13, 5593. https://doi.org/10.3390/su13105593
42. Lally, P.; van Jaarsveld, C. H. M.; Potts, H. W. W.; Wardle, J. How Are Habits Formed: Modelling Habit Formation in the Real World. European Journal of Social Psychology 2010, 40 (6), 998–1009. https://doi.org/10.1002/ejsp.674.
43. Ličen, S.; Prosen, M. Strengthening Sustainable Higher Education with Digital Technologies: Development and Validation of a Digital Competence Scale for University Teachers (DCS-UT). *Sustainability* 2024, *16* (22), 9937. https://doi.org/10.3390/su16229937.
44. Howard, S. K.; Tondeur, J. Higher Education Teachers' Digital Competencies for a Blended Future. Educational technology research and development 2023, 71 (1), 1–6. https://doi.org/10.1007/s11423-023-10211-6.
45. Marín, V.I.; Castañeda, L. Developing Digital Literacy for Teaching and Learning. In Handbook of Open, Distance and Digital Education; Zawacki-Richter, O., Jung, I., Eds.; Springer Nature: Singapore, 2022; pp. 1–20. ISBN 978-981-19035-1-9.
46. Gutiérrez-Ángel, N.; Sánchez-García, J.-N.; Mercader-Rubio, I.; García-Martín, J.; Brito-Costa, S. Digital Literacy in the University Setting: A Literature Review of

Empirical Studies between 2010 and 2021. Frontiers in Psychology 2022, 13. https://doi.org/10.3389/fpsyg.2022.896800.
47. Audrin, C.; Audrin, B. Key Factors in Digital Literacy in Learning and Education: A Systematic Literature Review Using Text Mining. Education and Information Technologies 2022, 27. https://doi.org/10.1007/s10639-021-10832-5.
48. Fernández, A.; Gómez, B.; Binjaku, K.; Meçe, E. K. Digital Transformation Initiatives in Higher Education Institutions: A Multivocal Literature Review. Education and Information Technologies 2023, 28. https://doi.org/10.1007/s10639-022-11544-0.
49. Zhao, Y.; Llorente, A. M. P.; Gómez, M. C. S. Digital Competence in Higher Education Research: A Systematic Literature Review. Computers & Education 2021, 168 (168), 104212. https://doi.org/10.1016/j.compedu.2021.104212.
50. Timotheou, S.; Miliou, O.; Dimitriadis, Y.; Sobrino, S. V.; Giannoutsou, N.; Cachia, R.; Monés, A. M.; Ioannou, A. Impacts of Digital Technologies on Education and Factors Influencing Schools' Digital Capacity and Transformation: A Literature Review. Education and Information Technologies 2022, 28 (28), 6695–6726. https://doi.org/10.1007/s10639-022-11431-8.
51. Saltos-Rivas, R.; Pavel Novoa-Hernández; Rocío Serrano Rodríguez. Understanding University Teachers' Digital Competencies: A Systematic Mapping Study. Education and Information Technologies 2023, 28. https://doi.org/10.1007/s10639-023-11669-w.
52. Jiang, X., Shi, D., Topps, A. K., & Archer, C. M. From Family Support to GoalDirected Behaviors: Examining the Mediating Role of Cognitive Well-Being Factors. Journal of Happiness Studies, 2019. https://doi.org/10.1007/s10902-01900117-7
53. Chen, Z. J. Unswervingly Promote the Development of Vocational Education Types in China Along the New Road of Chinese-style Modernisation. China's Education News, 2021. http://www.moe.gov.cn/jyb_xwfb/moe_2082/2021/2021_zl47/202108/t20210804_548835.html
54. Wu, X.; Ye, Y. Curriculum of Technical and Vocational Education. Technical and Vocational Education in China 2018, 61–95. https://doi.org/10.1007/978-981-130839-0_3.
55. United Nations. THE 17 GOALS. Sustainable Development. Available online: https://sdgs.un.org/goals (accessed on 12 August 2024).
56. Yu, S. A Research on University Students' Behavioral Intention to Use NewGeneration Information Technology in Intelligent Foreign Language Learning. ACM Transactions on Asian and Low-Resource Language Information Processing 2022. https://doi.org/10.1145/3563774.
57. Dang, H. L. An Analysis of the Factors Affecting Intention to Use Artificial Intelligence Technology in Learning: A Case Study of Hanoi Students. AJMI - ASEAN Journal of Management and Innovation 2020, 7 (2), 1–16. https://ajmi.stamford.edu/index.php/ajmi/article/view/183
58. Chatterjee, S.; Bhattacharjee, K. K. Adoption of Artificial Intelligence in Higher Education: A Quantitative Analysis Using Structural Equation Modelling. Education and Information Technologies 2020, 25 (5), 3443–3463. https://doi.org/10.1007/s10639-020-10159-7.

59. Michie, S.; van Stralen, M. M.; West, R. The Behaviour Change Wheel: A New Method for Characterising and Designing Behaviour Change Interventions. Implementation Science 2011, 6 (42). https://doi.org/10.1186/1748-5908-6-42.
60. NICE. Overview | Behaviour change: Individual Approaches | Guidance | NICE. Nice.org.uk. https://www.nice.org.uk/guidance/PH49.
61. Michie, S.; Atkins, L.; West, R. The Behaviour Change Wheel: A Guide to Designing Interventions; Silverback Publishing: London, 2014.
62. Keyworth, C.; Epton, T.; Goldthorpe, J.; Calam, R.; Armitage, C. J. Acceptability, Reliability, and Validity of a Brief Measure of Capabilities, Opportunities, and Motivations ("COM-B"). British Journal of Health Psychology 2020, 25 (3), 474–501. https://doi.org/10.1111/bjhp.12417.
63. Leahy, D.; Dolan, D. Digital Literacy: A Vital Competence for 2010? Key Competencies in the Knowledge Society 2010, 210–221. https://doi.org/10.1007/978-3-642-15378-5_21.
64. Nkosikhona Theoren Msweli; Tendani Mawela; Hossana Twinomurinzi. Transdisciplinary Teaching Practices for Data Science Education: A Comprehensive Framework for Integrating Disciplines. Social sciences & humanities open 2023, 8 (1), 100628–100628. https://doi.org/10.1016/j.ssaho.2023.100628.
65. Daniela Musicco Nombela; Dominici, P.; Sarasqueta, G.; Ma José Gato; Ma Joao Silveira; José Díaz Cuesta. Nueva Educación Universitaria En Línea: De Lo Emocional a La Espectacularización. Revista Latina de Comunicación Social 2023, No. 81, 509–538. https://doi.org/10.4185/rlcs-2023-1980.
66. Benvenuti, M.; Cangelosi, A.; Weinberger, A.; Mazzoni, E.; Benassi, M.; Barbaresi, M.; Orsoni, M. Artificial Intelligence and Human Behavioral Development: A Perspective on New Skills and Competences Acquisition for the Educational Context. Computers in Human Behavior 2023, 148, 107903. https://doi.org/10.1016/j.chb.2023.107903.
67. González López Ledesma, A. E. Las Competencias Digitales En El Currículo Argentino de Educación Digital. IE Revista de Investigación Educativa de la REDIECH 2022, 13, e1275. https://doi.org/10.33010/ie_rie_rediech.v13i0.1275.
68. García, B. M. El Desarrollo de La Competencia Digital En La Normativa Curricular Española. Opción 2015, 31 (1), 828–850. https://www.redalyc.org/articulo.oa?id=3104300504
69. Julca, C.; Rosa, L.; Soledad, K.; Maximiliana Quispe Gómez. Desarrollo de Competencias Digitales En Docentes de La Universidad Nacional Santiago Antúnez de Mayolo En Tiempos de Covid-19. Horizonte de la ciencia 2022, 12 (23). https://doi.org/10.26490/uncp.horizonteciencia.2022.23.1469.
70. Montero, C.; Hortensia, B.; Velez, P.; Montero, F.; Esperanza, V. Estrategias de Aprendizaje Virtual Y Competencia Digital En Estudiantes de Una Universidad Pública de Lima, 2022. https://www.redalyc.org/journal/3606/360674839009/360674839009.pdf
71. Pérez García, E. A.; Andrade Cázares, R. A. Orientación de La Competencia Digital Del Profesor Universitario En Las Propuestas de Integración de TIC. IE Revista de Investigación Educativa de la REDIECH 2020, 11 (0), e905. https://doi.org/10.33010/ie_rie_rediech.v11i0.905.
72. Romero, M.; María Soledad Ramírez-Montoya; Mariana Buenestado Fernández; Fernando Lara Lara. Use of ChatGPT at University as a Tool for Complex

Thinking: Students' Perceived Usefulness. Journal of New Approaches in Educational Research 2023, 12 (2), 323–323. https://doi.org/10.7821/naer.2023.7.1458.
73. Nursetiawati, S. Analysis Validity and Reliability as an Instrument to Measure the Self-Academic Concept in a College Student of Cosmetology of Engineering Faculty, State University of Jakarta. KnE Social Sciences 2019, 3 (12), 297. https://doi.org/10.18502/kss.v3i12.4095.
74. Hair, J. F., Black, W. C., Babin, B. J., & Anderson, R. E. Multivariate Data Analysis: A Global Perspective, 7th Edition. 2010, New Jersey: Pearson Educational Inc.
75. UNESCO Insitute for Lifelong Learning. 4th GLOBAL REPORT ON ADULT LEARNING AND EDUCATION.(2021). https://unesdoc.unesco.org/ark:/48223/pf0000377912?posInSet=29&queryId=Na0c2fcda-07bf-4ff4-a86d-27b487414db1
76. UNSD Progress Chart. The United Nations Statistics Division (UNSD) (2023). Progress Chart—SDG Indicators. Available online: https://unstats.un.org/sdgs/report/2023/progress-chart/ (accessed on 28 June 2024).
77. National Bureau of Statistics of the People's Republic of China. "第七次全国人口普查."[Seventh National Population Census]. Stats.gov.cn, 2021, www.stats.gov.cn/zt_18555/zdtjgz/zgrkpc/dqcrkpc/ggl/202302/t20230215_1904002.html.
78. Kenny, D. A.; McCoach, D. B. Effect of the Number of Variables on Measures of Fit in Structural Equation Modeling. Structural Equation Modeling: A Multidisciplinary Journal 2003, 10 (3), 333–351. https://doi.org/10.1207/s15328007sem1003_1.

Supporting Information

Click here to access/download
Supporting Information
software.rar
