# Supplementary material for: Artificial intelligence literacy, sustainability of digital learning and practice achievement: A study of vocational college students
Source: PLoS One. 2025 Oct 1;20(10):e0332175. doi: 10.1371/journal.pone.0332175 (PMC12488029; doi:10.1371/journal.pone.0332175)
Supplement: Attachment — Submitted filename: Reply to the article revision suggestions3.docx [file pone.0332175.s005.docx]

**Response to Reviewer Comments**

Dear Editor and Reviewers,

We sincerely thank you for your thoughtful and constructive comments on our manuscript entitled “**Artificial Intelligence Literacy, Sustainability of Digital Learning and Practice Achievement: A Study of Vocational College Students**”. We appreciate the time and effort you have invested in reviewing our work.

We have carefully considered all the comments and suggestions provided by the reviewers and have revised the manuscript accordingly. Below, we provide a detailed, point-by-point response to each comment.

Firstly, we have categorized and summarized the comments from Reviewer 1 and identified seven points for revision.

(R11)Your study effectively highlights the impact of AI on education, particularly in vocational higher education, but some areas could be clearer. The introduction is broad, and specifying what kind of attention vocational education lacks—whether in research, policy, or institutional support—would strengthen your argument.

(R12)The mention of the COM-B model is useful, but briefly explaining its focus on capability, opportunity, and motivation would help unfamiliar readers.

(R13)Your methodology is clear, but specifying whether the institution studied is a vocational college would enhance precision.

(R14)Some findings could be more clearly articulated, such as the statement that AI literacy correlates with sustainable digital learning behaviors through the sustainability of digital learning competences, which could be simplified for better readability.

(R15)Additionally, the claim that students in vocational institutions struggle to translate learning behavior into good performance in practice needs further clarification—does this mean they lack application skills, institutional support, or workplace opportunities?

(R16)The idea that sustainability of digital learning competences can be “slightly counterproductive” is intriguing but needs more explanation; in what way does it hinder performance?

(R17)The connection to SDG5 is interesting, but it is not entirely clear how controlling for gender, family resources, and ethnicity contributes to educational equity—elaborating on this link would strengthen the conclusion. A clearer and more precise articulation of these key points would enhance the impact of your study.

Similarly, Reviewer 2's comments are listed below in a point-by-point format.

(R21)Topic: Artificial Intelligence Literacy, Sustainability of Digital Learning and Practice Achievement: A Structural Equation Model. There is need to align the topic with the topic. The use of analytical tool on the topic is not necessary but making the topic to cover contents and context will be of a great value and attractive to the readers.

(R22)Introduction: “To enable better use of AI, data science education and data literacy may need to be integrated into K-12 education. Considering the context of higher education and TVET in the opening statements, what is the rationale for introducing the use of AI, data science education, and data literacy into K-12? As Chiu [12] suggests, the relationship between the two is currently not well-tested. What the two in this context?

(R23) Problem Statement: Problem statement is vague. There is need for critical analysis of literature to identify the gaps for the study.

(R24)Research Questions: RQ1: Is there a correlation between AI literacy and sustainable digital learning competences? This is good but no background speaks to the dependent variable (sustainable digital learning competence) Please include this.

RQ2: To what extent does AI literacy and the sustainable digital learning competences affect students' learning behaviors? Does this behavior facilitate or help students translate it into practical skills? At beautiful as these questions are, they are double barrel, please split them or transform them to a hypothesis to test the role of AI literacy and sustainable digital learning competences on students’ acquisition of practical skills. Also, provide background to this.

RQ3: Can AI literacy and sustainable digital learning competition contribute more to educational equity (SDG5) in a way? This is good. What is educational equity in the context of this study?

(R25)Literature: The concepts explained are good, however, most information provided could have been used at the study background.

“digital competence (DC) is interpreted in various ways (such as digital literacy, digital competence, electronic literacy, electronic skills, electronic competence, computer literacy, and media literacy)” This is not clear. There is repetitions here digital competence-digital competence, electronic competence; i think the focus should be what these digital competences are.

We adopt the above concepts and qualify them with the expectation that students will be able to use digital resources sustainably for a long period of time or with a high frequency while learning each skill. This is not clear, what concepts are above. What is learning competences, digital competence and sustainable digital learning competences? How are these measured in literature and specifically, in this study?

No concept clarifications for these: educational equity,

students' learning behaviors, and practical skills.

(R26)Theoretical Framework: The adoption of COM-B, but the proponent, year, justification, and application of it to current study is vague.

(R27)Hypothesis: It should be in plural form “Hypotheses” since they are more than one. Also, some of the hypotheses raised are not aligned with research questioned raised at the background. Also, empirical literature was not advanced for based on hypotheses formulated.

(R28)Framework: There is a spelling error ‘Farmework’ Also, is this conceptual framework or theoretical framework. The figure projects conceptual framework nor theoretical framework. There is need to elaborate how the framework summarises the study and supported by the theory adopted.

(R29)Ethical Consideration: Move it under the Methodology

(R210) Methodology: The explanations and presentation are good. However, technically and logically, there are areas for further explanations. What research philosophical and design adopted for the study and rationale for the adoption? What is the population of the study area and why is the adoption of convenience sampling approach? How is the sampling technique adopted provide protect the data and findings from bias? On the questionnaire, was the questionnaire adopted, adapted or researchers developed?

(R211)Questionnaire: “Investigate students' AI literacy and digital learning skills, and a five-point Likert scale” Where is sustainable digital learning competences, learning behavior, and Practice Achievement? How the data was collected (Online or paper) is missed. The construct “Practice Achievement” What the research has to say here is confusing, which tending towards quasi-experimental approach.

(R212)Analysis: What is the correlation between “Fig3. SEM of AI Literacy, Sustainable Digital Learning Competencies and Behavior” and “In Model 3, AI literacy and sustainable digital learning competencies and practice achievement”

The study background and literature review did not provide basis for this model. Now, the model tends towards negative, why must the model be retained and explained? The figure for control variables is invisible in the report. No hypotheses or research questions that support the control variables?

(R213)Discussion: The discussion was not presented logically based on the research hypotheses formulated. Furthermore, the findings were not discussed to establish the relationship that exist between the variables and the implications.

(R214)Conclusions, Implications and Limitations: The conclusion provided is fair. “This study examined the relationship between AI literacy, sustainable digital learning competencies, students' digital learning behaviors and practical performance with 1004 senior students in Liaoning Province.” However, the introduction of concepts such as students’ digital learning behaviors and practical performance are posing confusion to the readers. The implications of study findings and limitation are missing which are very necessary for study of this type.

Similarly, the annotations provided by the editor within the manuscript are listed below.

Commented [OS1]: The first sentence is a bit broad. You might clarify how AI's rise is specifically affecting education rather than stating it as a general trend.

Commented[OS2]: Your discussion effectively highlights the challenges and opportunities AI presents in education, particularly in the context of SDG 4. However, some areas could be more concise and precise. The link between AI and inequities in education is well stated, but phrases like “disable some of the ability of students to learn” could be clearer—does this refer to over-reliance on AI or gaps in digital literacy? Additionally, the mention of ethics and inequity could be further connected to specific risks, such as algorithmic bias or accessibility issues. The argument about the lack of rigorous rubrics to assess AI’s impact is strong, but specifying what metrics or frameworks might be needed would add depth. The transition to China’s higher vocational education is relevant, but stating why this group is “not in the mainstream” would

provide clarity. Overall, refining phrasing and strengthening logical connections between AI literacy, sustainable learning, and educational equity would improve the clarity and impact of the section

Commented [OS3]: Your discussion provides a thorough overview of AI literacy, but some areas could be more concise and precise. The definition section is well-supported by literature, but phrases like “educating learners to acquire fundamental concepts, skills, knowledge, and attitudes that do not require a priori knowledge” could be clearer—does this mean AI literacy is accessible without prior expertise? The transition from K-12 to higher education is strong, but clarifying how vocational education specifically fits into this continuum would enhance clarity

Commented [OS4]: Improve clarity, reducing redundancy, and reinforcing logical connections would strengthen this section.

Commented [OS5]: Your discussion effectively contrasts different theoretical models for studying AI adoption and sustainable digital learning behaviors, but it could be more concise.

Commented [OS6]: Your discussion effectively traces the evolution from digital literacy to AI literacy, highlighting their interconnectedness. However, the transition between concepts could be more fluid, particularly in linking digital competence and AI’s role in education. The engineering education example is relevant but could be more concisely integrated to strengthen the argument. The necessity of AI literacy in vocational education is well justified, but reducing

redundancy would improve clarity. The hypothesis (H1) is logical and well-grounded in the preceding discussion.

Commented [OS7]: The statement effectively conveys the reliability and validity of the questionnaires, as well as the strong model fit. However, it could briefly mention any key implications or limitations to provide a more comprehensive conclusion.

We would like to once again express our sincere gratitude to the reviewers and editors for their valuable feedback and efforts. We have summarized all the revision suggestions and integrated the common issues. Our responses are presented below. In the tracked version, some newly added references have led to higher-numbered citations appearing earlier in the text; this will be corrected in the final clean version.

**Title:**

**(R21)** Doubts about the readability of titles.

To enhance the readability of the title and avoid research tools appearing in the title, change the title to:

AI Literacy and Sustainability of Digital Learning: Can Vocational Students Translate These into Practical Achievements?

**Abstract:**

**Commented [OS1]** Doubts about the scope being too broad

The scope of the first sentence is indeed too broad. Please make the following modifications:

The rapid expansion of AI and the massive use of digital learning are creating a huge change in higher education. In contrast to general higher education, which is at the center of change, the changes in vocational higher education do not seem to have received sufficient attention from researchers.

**Introduction:**

**(R11)**

We fully agree that clarifying the specific aspects in which vocational education has been under-addressed would improve the precision and argumentative strength of the introduction. In response, we have revised the introduction to explicitly identify three key dimensions where vocational education remains insufficiently supported:

1.Research dimension: Most existing studies on AI literacy and digital learning focus on general education or higher academic institutions. Vocational education, especially concerning marginalized student groups, remains underrepresented in empirical research.

2.Policy resource allocation: Compared to comprehensive universities, vocational institutions often have limited access to digital infrastructure, educational resources, and faculty development opportunities related to AI and digital learning.

3.Institutional support: There is a noticeable absence of systematic integration of AI tools and data literacy into the curriculum in vocational education, which constrains students' ability to develop sustainable digital learning competencies.

These revisions have been incorporated into the introduction, and we have also improved the connection between this background and our stated research questions. We sincerely appreciate your insightful suggestion, which helped us improve the clarity and relevance of our research focus.

**(R22)**

We fully understand your concern about the logical coherence and the scope of the study. Upon careful consideration, we agree that this part is not closely related to the main focus of our research, which lies in the context of higher education and vocational education. To maintain the clarity and coherence of the manuscript, we have decided to remove this section entirely. Additionally, the ambiguity related to the citation of Chiu [12] has also been resolved as a result of this deletion. We sincerely appreciate your thoughtful feedback, which has helped improve the focus and rigor of our work.

**(R23) (R24)** Argument and explanation of research questions

We sincerely thank the reviewer for this insightful comment. In the revised manuscript, we have significantly strengthened the problem statement by integrating a more comprehensive and critical review of existing literature across several educational contexts—K-12, higher education, and vocational education. Specifically: Literature Mapping and Gap Identification: We systematically traced the evolution of AI literacy from digital literacy and identified its multidimensional nature as conceptualized by UNESCO and Ng. While prior studies have investigated AI literacy in K-12 and university settings (Ng [13], Druga et al. [16], Long et al. [22]), we highlighted the limited attention to vocational education, particularly in under-resourced environments. This lack of empirical focus on vocational students represents a concrete research gap. Theoretical and Practical Significance: Building on the existing AI literacy frameworks, we introduced the emerging construct of sustainable digital learning competence and supported it with references from ICILS, OECD, and recent empirical models (Ličen [43]; Yang [41]). We demonstrated that while digital competence and learning competence are increasingly essential in educational success, there is inadequate empirical work connecting these constructs to behavioral outcomes in vocational settings. Contextual Relevance and Marginalization: We expanded the discussion of educational inequality, emphasizing how vocational students in China are structurally marginalized in terms of institutional resources, societal perceptions, and curriculum design. These factors amplify the significance of our study within the Sustainable Development Goals (SDGs) framework, particularly regarding SDG 4, SDG 5, SDG 9, and SDG 10. Explicit Research Questions: Finally, to further enhance clarity and logical progression, we explicitly articulated three research questions (RQ1–RQ3) to examine the relationship between AI literacy, sustainable digital learning competence, and educational equity. We believe these improvements directly address the reviewer's concerns and clarify the scholarly contribution of our study. The revised introduction now provides a logically coherent, literature-grounded, and policy-relevant problem statement that aligns with the journal’s focus on contemporary educational challenges in the digital era.

**Literature Review**

**(R17)(R25)**Doubts and Explanations on the Definition of Concepts such as Digital Competence and Educational Equity

Clear definitions have been provided for learning ability, digital ability, and sustainable digital learning ability. The paragraph has been rewritten and deleted. The situation is as follows:

Sustainability digital learning competencies need to be defined in an aggregated manner in three dimensions, namely digital competence, learning competencies and sustainability behaviours. Digital Competence (DC) has been interpreted in a number of ways [33], with the International Institute for Computing and Information Literacy (ICILS) definition of digital competence as an individual's ability to use computers for investigative, creative, and communicative purposes in order to participate effectively at home, school, workplace, and in the community being commonly used. Learning competence is the way in which individuals (and organisations as groups of individuals) identify, assimilate and use knowledge [38]. It is widely recognised in the academic community that learning competence consists of three elements: goals (motivation), will and ability (knowledge and practice) [40]. Aggregating the above two definitions yields a definition of digital learning competence repeatedly called for by several organisations such as UNESCO and OECD [34], defining digital learning competence as a set of knowledge, skills and attitudes that enable students to use digital tools effectively for learning in digital learning environments [41]. Sustainable behaviours are behaviours that a person adopts over a long period of time or periodically, which are often referred to as ‘habits’ by psychologists [42]. A student is considered to have sustainable behaviour if he/she is able to adopt a certain behaviour for more than 21 consecutive days. Aggregating the above concepts leads to the definition of sustainable digital learning competence, which is the ability to use digital resources over a long period of time or frequently to effectively use digital tools for learning in a digital learning environment.

Digital literacy is a critical and evolving competency that goes beyond the basic use of digital tools. For example, proficiency in the use of digital tools, integrating digital simulations, and facilitating interactions between students through digital platforms are becoming increasingly important in modern education [43,44]

Defined and expressed the concept of educational equity:

Specifically, educational equity in SDG is seen as equality of ability, or a real opportunity to realize the function of education [80,81], but this does not seem to be well achieved. According to the PISA [81] survey with previous studies, student achievement varies across family socioeconomic status (SES), gender, and across ethnicity [82,83,84]. For example, family social status (SES) had different levels of influence on different subjects, with r=0.202 for math, while verbal achievement accounted for r=0.271. gender differences [85] gave females the upper hand in verbal achievement, but the academic ability gap was not significant. In contrast, differences in the performance of ethnic minority student groups have received attention in the last century, and this group tends to have a more disadvantaged socioeconomic status and distinct cultural characteristics, and it has been demonstrated that ethnic minority students are at a disadvantage in terms of performance compared to majority students [86].

In conclusion, in order to promote equity (SDG 5,SDG 10), it is hoped that factors such as family social status, gender, and ethnic minorities do not have an impact on students' digital learning behaviors and practical achievements.

However, for general terms such as practical skills and learning behaviors, we will not provide too much description and definition. The study of these terms has accurate and general meanings, and there is no need to define them. However, considering the flaws in the evaluation criteria for practical skill achievements in the text, we have made modifications and re descriptions in (R11).

**(R12)(R26)(R28)** COM-B Model and Framework

Both reviewers have raised doubts about the introduction of the COM-B model in the article, and we are very grateful for this. So we rewrote this part and made revisions to the generation and application of the COM-B model, as well as the exercises in this article. It is as follows:

There are a number of models to consider in the study of sustainable digital learning capabilities and students' sustainable learning behaviours. For example, the Technology Acceptance Model (TAM) Unified theory of acceptance and use of technology (UTAUT) have both been applied to the study of students' intentions towards AI. These models have been widely acted on undergraduate student population by researchers. For example, Le Dang [57] in analysing the intentions of university students to use AI for learning, Yu [56] in studying the behavioural intentions of university students to use next-generation information technology, including AI, in intelligent foreign language learning, and Chatterjee and Bhattacharjee [58] in investigating the effects of behavioural intentions on AI adoption in higher education. It seems that both models have very relevant capabilities to undertake this task, but all of them share a rather interesting point: it is not possible to analyse all independent variables by integrating them into a single capability or a single literacy in a comprehensive consideration and behaviour. Therefore, we wanted to find a model that could address this issue at a larger or more macro level. Not only that, but these two models can only study behavioural intentions, not behaviour itself, and for this reason the COM-B model was introduced.

COM-B aims to provide an overarching model that encompasses all the factors known to influence behaviour change [59]. The model was proposed by the UK's National Institute for Health and Care Excellence (NIHCE) in 2011, citing the Capabilities, Opportunities, Motivations, Behaviours (COM-B) model [59] as a key theoretical framework for understanding and supporting behaviour change [60]

And, in order to better understand the connection between the theoretical framework and the conceptual framework, we have redrawn the **Fig1**.

**(R27)** Hypothesis

We have transformed it into the plural form of the hypothesis.

**Commented [OS3],Commented [OS4],**

These opinions mainly revolve around language conciseness and coherence. Therefore, we have made some deletions to this part of the content and enhanced its coherence and readability. For example,[OS3]:

Based on Ng's [13] description of awareness and impact, the 27 article conceptualises AI literacy in the following form: educating learners to master the concepts, skills, knowledge and attitudes underlying knowledge, but without any a priori knowledge base. In vocational education, based on the sparse existing literature, Ferhat [79] et al. combined the nursing profession with AI to integrate academic support mechanisms into the digital transformation process of educational programmes.The work done by Ferhat appears to be in a state between the simple situational interactions of k-12 and the specific complex interactions of higher education, whereby the teacher performs the appropriate pedagogical interventions in anticipation of the student completing the specific and paradigmatically fixed operational processes.

**Commented [OS5],Commented [OS6]**

Similar to the commented [OS3] and commented [OS4] above, we hope to make the discussion concise and efficient. We have made some deletions to make our argument more readable and coherent.

**R(14)**

Similar to the above Commented [OS5] and Commented [OS6], we hope to make the discussion concise and efficient. To this end, we have made some deletions to make our argument more readable and coherent.

**Methodology**

**(R29)**Ethical Consideration: Move it under the Methodology

Modifications have been made

**(R13)**Suggestions for sample selection

In the newly revised version, we have placed greater emphasis on the selection criteria for school departments and the basic information about the vocational college.

**(R210),(R211)**Explanation of sample selection methods and institution selection methods

We have made adjustments to the selection of institutions, and the revised version is as follows:

A total of 1004 first year students (male = 869; female = 134) from four faculties of a higher vocational and technical school in Lioaning with a mean age of 19.93 years (SD = 2.645) participated in this study.

In order to ensure the objectivity of the study, the researcher will not be directly involved in daily teaching and learning, and will only make suggestions on the curriculum design of AI use and digital learning for frontline teachers' enquiries. As there are not yet many schools in the region that have carried out instructional design on the curriculum of AI use and digital learning, only this vocational institution can be used for the study.In order to ensure the reliability of the data for the study, all students who participated in this course on AI use and digital learning were included in the study as a sample. To protect the privacy of the students, anonymity was used to fill in the data collection.

The sample selection and measurement methods have been readjusted, and the modified results are as follows:

In order to ensure the objectivity of the study, the researcher will not be directly involved in daily teaching and learning, and will only make suggestions on the curriculum design of AI use and digital learning for frontline teachers' enquiries. As there are not yet many schools in the region that have carried out instructional design on the curriculum of AI use and digital learning, only this vocational institution can be used for the study. In order to ensure the reliability of the data for the study, all students who participated in this course on AI use and digital learning were included in the study as a sample. To protect the privacy of the students, anonymity was used to fill in the data collection.

The questionnaire was designed through Questionnaire Star (https://www.wjx.cn/), which consisted of thirty-three questions, of which the ten questions in the first section were basic information about the individual, which contained some basic variables that needed to be controlled。

The specific calculation method is shown in formula 1 below:

$x_{score}=\frac{1}{3}\sum_{i=1}^{3} x_{i}$ $0\leq x_{i}\leq100$ (1)

$x_{i}$is an independent rating for each teacher.

**Discussion**

**(R15)(R212)** Some replies and analysis on the model's tendency towards negative results.

We made moderate modifications in the discussion section in order to address these issues.

This view is supported by some local school teachers, who say that the current rise of AI does not have the ability to integrate digital resources, making it difficult for students to know exactly which resources are key and important.

This resistance does not stem from a lack of resources or learning opportunities, but purely from the students' own internal motivation.

Since the path of Model 3 is not significant, there is no need for specific analysis, so no stability analysis was performed. However, considering that Model 3 is included in the research hypothesis and plays an active role in the theoretical analysis, it was retained. This phenomenon is explained in the discussion.

**(R16)(R213)** Establishing the relationship between variables in the discussion

We have made minor adjustments to the discussion section and provided a reasonable explanation for how to hinder students from transforming their digital learning abilities into practical skills. The specific situation is as follows:

Another reasonable explanation requires further investigation by researchers. In previous studies [96,97], researchers have raised concerns about whether students will develop a negative reaction to the digital age. Furthermore, teachers' digital literacy has a strong correlation with the development of digital literacy, meaning that students' digital learning abilities and behaviors are constrained by teachers' digital literacy [97].

**(R214),Commented [OS7]**:Discussion on Limitations

To compensate for the lack of limitations, we have added the following explanation:

This study demonstrates a stable relationship between AI literacy and sustainable digital learning capabilities in terms of learning behavior and practical achievements. However, there are two limitations: first, the model has too few latent variables, making it impossible to establish a connection between the observed variables of AI literacy and sustainable digital learning capabilities. Second, when measuring sustainable digital learning abilities, relying solely on four AI application courses to enable students to transform sustainable learning abilities into learning behaviors and further into practical abilities is not feasible. Additionally, similar AI literacy courses have not yet been fully implemented for vocational college students, making it impossible to conduct large-scale intervention teaching and measurement. As AI becomes more widespread, more AI courses and teaching interventions can bridge the current curriculum gap.
